# Facets of Autophagy Based Unconventional Protein Secretion–The Road Less Traveled

**DOI:** 10.3389/fmolb.2020.586483

**Published:** 2020-12-09

**Authors:** Sreedevi Padmanabhan, Ravi Manjithaya

**Affiliations:** ^1^Autophagy Laboratory, Molecular Biology and Genetics Unit, Jawaharlal Nehru Centre for Advanced Scientific Research, Bengaluru, India; ^2^Neuroscience Unit, Jawaharlal Nehru Centre for Advanced Scientific Research, Bengaluru, India

**Keywords:** autophagy, unconventional protein secretion, secretory autophagy, multivesicular body, exosome, GRASP

## Abstract

Unconventional protein secretion (UCPS) of leaderless proteins bypasses the conventional endoplasmic reticulum (ER)-Golgi route. The proportion of UCPS in the secretome varies tremendously across eukaryotes. Interestingly, macroautophagy, an intracellular recycling process that is generally involved in cargo degradation, also participates in UCPS. This emerging field of secretory mode of autophagy is underexplored and has several unanswered questions regarding the composition of players, cargo, and the mechanisms that drive it. As secretomes vary considerably across cell types and physiological conditions, the contribution of secretory autophagy in healthy and pathophysiological states remain to be elucidated. Recent studies have begun to shed light on this enigmatic process.

## Introduction

**FIGURE 1:**
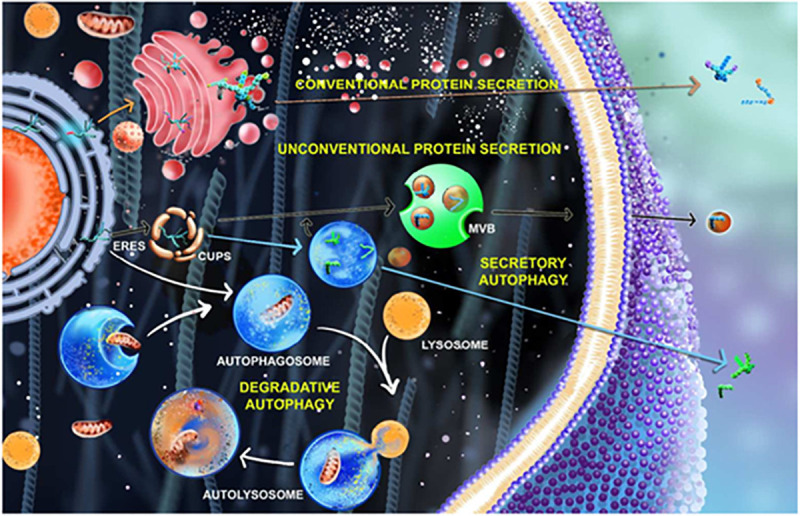
Secretory pathways in cell involving autophagy process. In the conventional protein secretion (marked by orange arrow), the proteins synthesized from ER containing the leader peptide undergoes post translational modifications in Golgi apparatus and gets secreted outside the cell whereas in unconventional protein secretion (marked by black and blue arrows). The proteins bypass the Golgi route or get trapped in CUPS near the ERES (ER exit site) and gets secreted outside that are mediated by autophagosomes (blue arrow) and multivesicular bodies (MVBs) (black arrow). The process of degradative autophagy involving phagophore expansion, autophagosome formation, lysosomal fusion, and degradation are showed using white arrow.

Secretion is a pivotal physiological process for the proper functioning of a cell, wherein the secretory proteins synthesized inside the cell are destined to be secreted out of the cell. The total secretory content (secretome) of many cells in the body of metazoans varies largely. However, certain human cells, such as endocrine cells and B-lymphocytes, are specialized for secretion of proteins. In conventional protein secretion (CPS), the ability of these proteins to be secreted is largely determined by the signal peptide. Pioneering work in the laboratories of James Rothman, Thomas Sudhof, and Randy Schekman, in elucidating the mechanisms underlying eukaryotic classical secretory pathway [endoplasmic reticulum (ER)-Golgi-secretory vesicles] demonstrated that proteins with signal peptides get secreted to the exterior and had (Hata et al., 1993; Sollner et al., 1993; Barlowe et al., 1994; Bonifacino, 2014; Viotti, 2016) led to the 2013 Nobel Prize in physiology and medicine (Figure 1). This canonical mode of protein secretion may be constitutive or regulated. During constitutive secretion, the proteins synthesized inside the cells are packaged into secretory vesicles and exocytosed, whereas in the regulatory mode, the proteins enveloped in the secretory vesicles or granules get secreted in response to relevant signaling cues.

The process by which proteins devoid of canonical leader sequence are secreted is termed as unconventional protein secretion (UCPS). Unlike the classical secretory proteins that follow the canonical route of secretion, the unconventionally secreted protein cargoes follow a plethora of divergent secretory mechanisms (Figure 1). Studies suggest the existence of four principal types of UCPS that can be further distinguished into non-vesicular and vesicular pathways (Rabouille et al., 2012; Rabouille, 2016). The non-vesicular pathways are further classified into Type I (e.g., FGF1) and Type II (e.g., yeast MATα). The vesicular pathways are mediated by Type III (e.g., Acb1) and Type IV (e.g., CFTR) mechanisms. Based on a recent classification, Type I is a pore-mediated translocation across the plasma membrane, Type II is an ABC transporter mediated secretion, Type III is an autophagosome/endosome-based secretion and Type IV is a Golgi bypass mechanism (Rabouille, 2016).

## Autophagy Dependent Secretion of UCPS Cargoes and Their Regulation

Autophagy is a conserved process of cellular recycling in eukaryotes. The autophagy proteins (ATG proteins) orchestrates the initiation, nucleation, expansion, maturation, fusion, and recycling processes bringing about homeostasis in cells. The process of autophagy is mediated by packaging of cellular components (damaged and/or redundant organelles) in a double-membraned vesicle called autophagosomes. These vesicle-containing cargoes eventually fuse with lysosomes to form autolysosomes where the cargo is degraded and recycled. Thus, this form of autophagy is primarily degradative in nature. A set of 42 ATG proteins identified in yeast and mammals are involved in this process. Some of these are core ATG proteins that are indispensable whereas others are functional only under selective autophagic conditions depending on the cargo captured for degradation. A unique feature of the autophagy process is ability to form *de novo* vesicles, that have cargo specificity. One such selective form of autophagy that participates in UCPS is known as secretory autophagy (Jiang et al., 2013) wherein the cargo is secreted out instead of being degraded. A small subset of proteins has been proposed to be secreted out by the autophagy machinery in UCPS (Schotman et al., 2008; Duran et al., 2010; Manjithaya and Subramani, 2010, 2011; Manjithaya et al., 2010; Dupont et al., 2011; Gee et al., 2011; Nilsson et al., 2013; Murrow et al., 2015; Son et al., 2015, 2016; Kortvely et al., 2016; Nuchel et al., 2018).

It is intriguing to know as to how are the proteins that are subjected for degradation or secretion identified? The proteins destined for lysosomal degradation are marked by ubiquitination which triggers the sorting of the cargo proteins into the lumen of late endosomal multivesicular bodies (MVBs)/endosomes. MVB formation occurs when a portion of the endosome’s limiting membrane invaginates and buds into its own lumen. These intraluminal vesicles (ILVs) are degraded by the lysosomal hydrolases when MVBs fuse with the lysosomes. In some cases, these MVBs can fuse with the plasma membrane releasing the internal vesicles as exosomes (Bobrie et al., 2011). Autophagosome-endosome interaction is required for the maturation of autophagosome and there are several lines of evidence that supports MVBs as the prime fusion partners (Berg et al., 1998; Fader and Colombo, 2009).

As MVBs are found to mediate the autophagy process (Hirsch et al., 1968), an interesting example of MVB-mediated secretion was described for the UCPS of yeast Acb1 that is mediated by autophagy (Duran et al., 2010; Manjithaya et al., 2010). Genetic studies in yeast has revealed that the core machinery necessary for autophagosome formation is required for a UCPS cargo Acb1, suggesting that secretory autophagosomes must be formed. There seems to be a specificity of plasma membrane SNAP receptor SNARE, Sso1 in Acb1 secretion and not Sso2 (Duran et al., 2010). Across species, Acb1 secretion assay was used to identify a subset of genes required for UCPS of Acb1 along with the genes involved in autophagosome and peroxisome biogenesis. This suggests a diversion of vesicles from an autolysosomal fate toward the plasma membrane. The autophagosome mediated Acb1 secretion is shown to be Golgi reassembly stacking (GRASP) protein, GRASP and Bug1 dependent as demonstrated by Vivek Malhotra’s group (Duran et al., 2010). Acb1 secretion in yeast is found to be time-dependent either on nitrogen starvation or on induction of autophagy by rapamycin (Manjithaya and Subramani, 2011) and cell-density dependent. The Acb1 secretion is found to be higher around 3.5–4 h after starvation. ACBP release in mammalian cells on rapamycin induction was also demonstrated in primary astrocytes (Loomis et al., 2010). In *Dictyostelium discoideum*, the spore differentiation factor, SDF2 peptide which is the processed form of AcbA (yeast Acb1 homolog) is found to be involved in sporulation thus acting as signaling molecules (Anjard and Loomis, 2005).

Concurrent to the genetic studies in yeast which demonstrated that Acb1 is unconventionally secreted *via* vesicles (Duran et al., 2010; Manjithaya et al., 2010), Vivek Malhotra’s group also identified that these vesicles are captured in a novel compartment called CUPS (Compartment for UCPS) (Malhotra, 2013). An elegant study from their group has demonstrated the requirement of the endosomal sorting complexes (ESCRT-I, II, and III) for the UCPS of Acb1 in yeast. Also, they reported that CUPS are not MVBs as their formation is independent of ESCRT-0 and Vps4 (Curwin et al., 2016). Snf7 (Vps32) is found to be a key endosomal sorting complexes required for transport (ESCRT) regulator required for UCPS in eukaryotes that renders stability of CUPS.

In another study, Snf7 and transcriptional repressors such as RIM101 was shown to have a regulatory role in the fungal pathogenesis of *Cryptococcus neoformans* (Godinho et al., 2014). This is evident from the upregulation of RIM101-mediated stress response in human meningitis caused by *C. neoformans* (Chen et al., 2014). Adaptation to changing environment or external stimuli by modulating phenotype is a common feature observed in eukaryotes. Studies in the fungal pathogen, *C. neoformans* demonstrated that Acb1, homolog of ScAcb1 is secreted unconventionally and is found to mediate yeast to the invasive hypha transition (Xu et al., 2015). Here, secreted Acb1 acts as a signal molecule for the morphological transition/switch. It is evident that these unconventionally secreted proteins, acting as a quorum sensor in yeast, have secondary roles that are unrelated to their core function, and hence referred to as “moonlighting proteins.” It is interesting to note that involvement of MVBs in sequestering leaderless secretory proteins (LSPs) and their role in pathogen defense (An et al., 2006) have also been demonstrated in plants.

Although UCPS is considered Golgi-independent, the role of two GRASP proteins, GRASP55 and GRASP65 are shown to play a significant role in the secretion of some leaderless proteins (Kinseth et al., 2007; Schotman et al., 2008; Gee et al., 2011). One of the classical examples of unconventional secretory protein, IL1-β has been demonstrated to translocate into secretory vesicles that is mediated by autophagy, MVBs and GRASP55. The association of HSP90 with two KFERQ-like sequences within the mature portion of IL1-β resembles chaperone mediated autophagy (Zhang et al., 2015) and requires GRASP55, Rab8a, and MVB formation (Dupont et al., 2011). Similarly, the latent TGFB1, another example of UCPS, is found to interact with the GORASP2/GRASP55 and secreted in an autophagy dependent manner that is mediated by Rab8a (Nuchel et al., 2018).

Multiple lines of evidence demonstrate the interplay of autophagy and UCPS in clinical and physiological context. The GRASP dependent unconventional secretion of the cystic fibrosis transmembrane conductance regulator (CFTR) demonstrates physiological relevance of UCPS in the cystic fibrosis disease (Gee et al., 2011). The disease-causing mutation of CFTR (ΔF508) results in conventional exocytosis defects. *In vitro* and *in vivo* studies have shown that unconventional GRASP-mediated pathway can rescue the phenotypic defect in CFTR. Inactivation of Beclin-1 complex is observed in the CFTR mutations of cystic fibrosis patients (Maiuri and Kroemer, 2018; Maiuri et al., 2019). Phosphorylation of C-terminal of GRASP55 leads to dissociation of GRASP homomultimer complexes which is found to be an essential requisite in the unconventional secretion of CFTR (Kim et al., 2016).

Autophagy plays a central role in the removal of protein aggregates within neurons as seen in the diseases such as Alzheimer’s, Huntington’s, and Parkinson’s (Rubinsztein et al., 2012). Autophagy dependent secretion of the neurodegenerative disease-causing aggregates such as α-synuclein, β-amyloid and tau that are leaderless proteins seem to have significant implications in the disease pathology. Studies indicate that the aggregate proteins such as mutant huntingtin and TDP43 are captured by the core autophagic machinery due to their substrate selection ability and functional MVBs (Yamamoto et al., 2006; Filimonenko et al., 2007). Impairment of autophagosome-lysosome fusion promotes tubulin polymerization-promoting protein (TPPP/p25α) to secrete α-synuclein, the hallmark protein in Parkinson’s disease, in an unconventional manner (Ejlerskov et al., 2013). Tau, which is a cytoplasmic protein, thought to be released only from degenerating cells are released by unconventional secretion. The secretion mechanism of tau protein is vesicle associated (Simon et al., 2012) and is also found to be temperature dependent (Chai et al., 2012). Temperature-dependent release of full-length tau proteins is independent of the classical secretory pathway. Studies in inducible Chinese hamster ovary (CHO) cell lines demonstrate that the phosphorylated Tau is preferentially selected for secretion (Katsinelos et al., 2018). Secretion of β-amyloid aggregates formed in Alzheimer’s disease is also mediated by autophagy. Knock out studies in mice neuronal Atg7 was found to influence the β-amyloid secretion, thereby affecting the plaque formation, a pathological hallmark of Alzheimer’s disease (Nilsson et al., 2013).

Insulin degrading enzyme (IDE), is a major endogenous Aβ-degrading enzyme that mediates Aβ clearance and is found to be secreted by unconventional secretory pathway. This enzyme is found to be secreted through autophagy-based unconventional secretion upon statin induction (Son et al., 2015) and has relevance in Alzheimer’s disease (Son et al., 2016). The IDE secretion from primary astrocytes was increased by statins in a time and concentration dependent manner mediated by autophagy, GORASP1 (GRASP65), GORASP2 (GRASP55), and Rab8a proteins (Son et al., 2016).

Moreover, depletion of an essential kinase PIKfyve by an inhibitor (Apilimod) leads to decreased autophagic flux, promoting the excess secretion of autophagy associated proteins such as NBR1, p62, OPTN, CACO2, and membrane bound LC3B in the form of vesicles (Hessvik et al., 2016). Reduced expression of antimicrobial peptides such as angiogenin 4, interlectin 1, and relmβ in the ATG7 knockdown led to reduction in mucin release that resulted in ulcerative colitis infection (Tsuboi et al., 2015). ATG5 and ATG16L1 deficiency also leads to reduced mucin (MUC5AC) release in the human tracheal epithelial cells (Dickinson et al., 2016). Exclusive dependency of ATG16L1 in neuropeptide Y (NPY) secretion (Ishibashi et al., 2012) and regulation of secretion of von Willebrand factor during vascular injury by ATG7 (Torisu et al., 2013) demonstrated clearly that the protein secretion is mediated by autophagy and thus play significant physiological roles. Autophagy proteins Atg5, Atg7, Atg4B, and LC3 are demonstrated to play significant role in polarized secretion of lysosomal contents in osteoclastic bone resorption and also in osteoporosis (DeSelm et al., 2011; Yin et al., 2019). These studies clearly demonstrate the process of UCPS in the normal functioning of a cell and points toward its relevance under disease conditions. The role of secretory autophagy in the context of various metabolic and degenerative diseases that warrants the need for autophagy dependent therapeutic interventions are reported (Cotzomi-Ortega et al., 2018; New and Thomas, 2019; Gonzalez et al., 2020). The comprehensive list of the autophagy dependent unconventionally secreted cargoes is tabulated (Table 1).

**TABLE 1:** List of autophagy dependent unconventionally secreted cargoes.

**Cargo**	**Cell type/organism**	**References**
Acb1	Yeast	Duran et al., 2010; Manjithaya et al., 2010
IL1β	HEK293T, U20S, MEFs cell lines	Zhang et al., 2015
TGFβ1	Primary murine fibroblasts	Nuchel et al., 2018
CFTR	HEK293	Noh et al., 2018
IDE	Primary astrocytes	Son et al., 2015; Son et al., 2016
P62, CAC02, NBR1, OPTN	Human prostate cancer epithelial cell line PC-3	Hessvik et al., 2016
α-SNCA	Rat pheochromocytoma cell line PC12	Ejlerskov et al., 2013
β-AMYLOID	Mice, primary cortical/hippocampal neurons	Nilsson et al., 2013
TAU	Primary cortical neurons	Mohamed et al., 2014
MUCIN (MUC5AC)	Primary human tracheal-bronchial epithelial cells (hTEC)	Dickinson et al., 2016
NPY	PC12 cells	Ishibashi et al., 2012
Von WILLEBRAND FACTOR	Human endothelial cells	Torisu et al., 2013
α-Crystallin B (or HSPB5)	HeLa, Cos7 cells	D’Agostino et al., 2019
FABP4	Differentiated adipocytes and primary adipocytes	Josephrajan et al., 2019

On a similar note, α-Crystallin B CRYAB or HspB5, a cardinal protein of vision in retinal cells are found to be secreted in an unconventional manner that is mediated by autophagy due to site specific phosphorylation at serine 59 residue (D’Agostino et al., 2019). Another example is the fatty acid binding protein 4 (FABP4) found in the adipocytes, which were thought to be secreted out in an unconventional manner that is GRASP independent (Villeneuve et al., 2018), but was found recently to be mediated by the process of autophagy (Josephrajan et al., 2019) in a sirtuin-1 dependent manner. Recent studies suggest that the ESCRT –associated protein Alix (PDCD6IP) is controlled by Atg12-Atg3 in the process of late endosome distribution, exosome biogenesis, and viral budding (Murrow et al., 2015). It is observed that Atg3 is an E2-like enzyme required for LC3 lipidation during autophagy and Atg12 is the conjugation target and is a ubiquitin-like molecule. The Alix protein has functional role in HIV-1 replication (Fujii et al., 2009).

Although trafficking of leaderless proteins leads to their secretion outside the cell by various means, only a subset of unconventional secretion is dependent on autophagy. Autophagy intersects with protein trafficking and secretion thus playing a broad role in the constitutive biosynthetic pathway (Narita et al., 2011), regulated exocytosis (DeSelm et al., 2011), and alternative routing of integral membrane proteins to the plasma membrane (Gee et al., 2011). The factors which may play a significant role and affect secretory autophagy such as time dependent, cell-density, temperature, post translational modifications, presence of MVBs, Rabs (GTPases), GRASPs, ESCRT, environmental stress/stimulus, transcriptional regulators and others such as Exocyst-Positive Organelle (EXPO) etc.

## Secretory Versus Degradative Autophagy – a Crosstalk?

**FIGURE 2:**
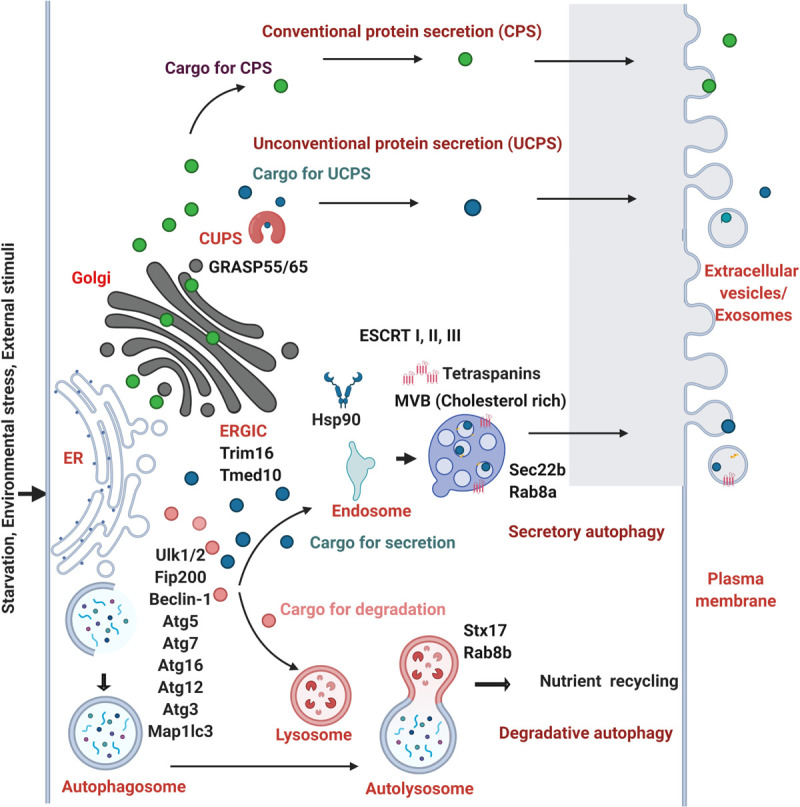
Molecular players involved in the crosstalk of autophagy and protein secretion. The cargoes of conventional protein secretion (CPS) (filled green circles) synthesized in the ER traverses the Golgi apparatus and are secreted out. The cargoes of unconventional protein secretion (UCPS) (filled dark blue circles) are secreted out in the absence of a leader peptide by passing through the ERGIC, TMED10, CUPS with the help of Hsp90 and Trim16 proteins. The process of autophagy is activated by the external cues such as starvation, environmental stress, stimuli that helps in the encapsulation of the cargo within the double membranous autophagosomes which fuses with the lysosomes forming autolysosomes in the degradative autophagy. The cargoes (filled marked in pink color) are degraded in the lysosomes. In the secretory autophagy process, a subset of secretory cargoes (dark blue circles) uses the common core ATG proteins such as Ulk1/2, Fip200, Beclin-1, Atg3, Atg5, Atg7, Atg12, Atg16, Map1lc3 (marked in the initiation steps of autophagy). *Multivesicular bodies* (MVBs) are formed from early endosomes by the inward budding of the limiting membrane into the lumen. MVBs that gets secreted by fusing with the plasma membrane are termed as exosomes, a form of extracellular vesicles. Distinct SNARE and Rab proteins are found to be involved in the selectivity of degradative (Stx17 and Rab8b) and secretory autophagy (Sec22b, Rab8a). The role of GRASP55 and GRASP65, HSP90, ESCRT I, II, III machinery proteins and tetraspanins are shown to play role in UCPS and secretory autophagy. The MVBs rich in cholesterol are shown to prefer the secretory mode.

Studies have shown that autophagy is induced by starvation, the structures formed within the cell, their dynamics, the involvement of PI3P. The induction of several autophagy related factors are common events both in secretory and degradative autophagy. Therefore, the question of how cells can distinguish and sort the cargo to delineate them into degradative or secretory routes of autophagy is of interest. At what stage and how is the fate of cargoes destined? Although these and other questions remain unanswered, there are a few clues that suggest that a crosstalk between the conventional and unconventional secretory routes. First, there is an involvement of Vps23 in yeast (Tsg101 in mammals), one of the partners in ESCRT-I complex in the process of formation of CUPS in secretory autophagy. At the same time, ESCRT-II and III proteins such as Vps25, Vps36, Vps20, and Vps2 are involved only in secretory autophagy rendering process specificity (Bruns et al., 2011). However, the role of autophagy adaptor proteins in the UCPS is not known. Secondly, MVBs are found to play a role in autophagy. There seems to be a coexistence of two different subsets of MVBs which might also play a role in decision making between the secretory and degradative autophagy. The cholesterol-rich MVBs are targeted for secretion whereas the cholesterol-poor MVBs are targeted for degradation (Mobius et al., 2002). The third factor comes from the clue that the biogenesis of CUPS is not triggered by rapamycin as in degradative autophagy. The fourth clue comes from the indications showing preferential recruitment of Rab8a in secretory autophagy to Rab8b in degradative autophagy (Pilli et al., 2012). The fifth clue comes from the environmental cues that determine the choice between the secretory versus degradative autophagy to maintain the energy balance of the cell. Although the destination of degradative autophagosome fusing with lysosomes and secretory autophagosomes fusing with the plasma membrane are different, biogenesis of these carriers, the cargo machinery may be common. However, the transport, tethers and SNAREs would be distinct for secretory and degradative autophagy. Recent report suggests the involvement of dedicated SNAREs and specialized TRIM cargo receptors that mediate secretory autophagy (Kimura et al., 2017). Finally, the concept of LC3 dependent EV loading and secretion (LDELS) from the secretomic studies has opened up more avenues to ponder upon the autophagy mediated protein secretory cargoes in detail (Leidal and Debnath, 2020; Leidal et al., 2020). From all the recent studies, it is clear that the initial core ATG proteins such as Ulk1/2, Fip200, Beclin-1, Atg5, Atg7, Atg16, Atg12, Atg3, and LC3 are pivotal in both the processes of degradative and secretory autophagy and the selectivity of the process is brought about by SNAREs, Rabs, and cholesterol enrichment (Figure 2).

## Current Research Gaps in the Field

The questions aimed at answering the mechanism that distinguishes secretory versus degradative cargo capture and delivery of the autophagosome to the correct destinations are key areas of ongoing research. The mechanistic insights into how vesicular mode functions are just beginning to unfold. In this vesicular mode, cargoes destined for secretion must be captured in vesicles and should eventually fuse with plasma membrane (and not lysosomes) to release the cargo to the exterior. Although there are some evidences of Rabs (Rab8a) and SNARE proteins (Sec22b) which are exclusive in this selective process of secretory autophagy, the role of other players is not well studied. It is also not clear whether the specificity of the cargo and the environmental cues play a significant role in autophagy dependent UCPS. Although there are lot of secretomic data available in the eukaryotic kingdom, not many autophagy mediated UCPS cargoes are reported and elucidated. It might be because these autophagy dependent cargoes are below the detection limits. This can be supported by the recent reports of ROS being a trigger for the secretion of many UCPS cargoes of which many of them are in very low abundance (Cruz-Garcia et al., 2020). It can also be speculated that autophagy might be a secondary mechanism of UCPS. PepN, an aspartic protease devoid of a leader peptide was found to be secreted in the filamentous fungus *Aspergillus niger* in an autophagy independent manner in carbon starvation conditions (Burggraaf et al., 2016). The UCPS triggered by the dectin-1 pathway is found to be a robust, autophagy dependent process and is dependent on the inflammasome activity (Ohman et al., 2014).

The main caveats in the field of UCPS (Based on the 1st UCPS meet at Assisi, 2019) are

1. Lack of common nomenclature amongst diverse secretory routes of UCPS
2. Identifying the substrates of each type of UCPS
3. Identifying the molecular mechanisms underlying the types of UCPS
4. Role of stress in the context of cell type specificity
5. Identifying membrane translocating proteins

The leaderless proteins are found to be more flexible than the leader peptide. Signal peptides are 15–50 amino acid tags that lead proteins destined for secretion. They have a tripartite structure – positively charged N-region, a hydrophobic H-region, and a polar C-region. It seems that the evolutionary pathways of the signal peptide differentiating between CPS and UCPS are distinct. Among mammals, analysis of the proteins in different subcellular compartments demonstrated that the secreted proteins evolve faster than the non-secreted proteins (cytosolic and nuclear) (Julenius and Pedersen, 2006). A comparison of the mouse and rat gene ortholog of secretory proteins suggests that signal peptides evolve five times faster than the flanking mature peptides (Williams et al., 2000). It is interesting to note that protein secretion is independent of tissue-specificity which means that the most rapidly evolving genes have a greater propensity to get expressed in fewer tissues. For example, brain specific secretory proteins evolve more slowly than secretory proteins from other tissues (Winter et al., 2004). Studies demonstrate that there is a relaxed positive selection in the signal peptides leading to faster evolutionary rates from prokaryotes (*Streptomyces coelicolor, Streptomyces avermitilis*) to eukaryotes (*Saccharomyces cerevisiae, Saccharomyces paradoxus*) (Li et al., 2009). This aspect of stronger evolutionary selection pressure over the non-secretory protein genes was further confirmed in symbiotic and pathogenic bacterial strains (Thakur et al., 2013). It is evident that in most of the Actinobacteria genomes studied so far, the ratio of non-synonymous (mutation) to synonymous substitutions (silent substitution) of the secreted proteins are stronger than the non-secretory proteins depicting that the secretory proteins evolve at a faster rate (Thakur et al., 2013). In a condition of inverse relationship between the evolution rate and the age of a gene and where the comparison is made with the exported proteins in unicellular yeast, the extracellular proteins are evolving faster. The possible reason suggested behind faster evolution of the extracellular proteins may be due to their relatively younger age during evolution and exon shuffling (Julenius and Pedersen, 2006). From an evolutionary perspective, all these above observations suggest that any instability may be easily accommodated in extracellular proteins as compared to intracellular counterpart. On similar grounds, secretome studies in *Caenorhabditis elegans* demonstrate that many members have no signal peptide in the large families of secreted proteins such as insulin but their physiological roles are not elucidated (Suh and Hutter, 2012). Although considerable amounts of leaderless proteins are secreted, the evolutionary forces that determine their secretion dynamics is not known. The recent identification of the single spanning membrane protein TMED10 (transmembrane p24 trafficking protein 10) as a novel component of the translocation machinery whose activity allows the recognition and loading of many cytosolic cargoes which are then secreted through unconventional secretion *via* ER-Golgi Intermediate Compartment, ERGIC (Liu et al., 2020; Zhang et al., 2020) opens up wide implications in understanding the molecular players.

## Key Research Perspectives

It is well known that multiple environmental signals such as pH, temperature, carbon source, host interactions, and regulatory factors influence the composition of the fungal secretome (McCotter et al., 2016). It is quite plausible that the secretory autophagy might be a type of selective autophagy pathway which orchestrates based on the environmental cues. There seems to be a consensus that ubiquitination is necessary for sorting proteins into ILVs destined for degradation through the fusion of MVB with lysosomes. On the contrary, unconventional secretion of cytosolic proteins could also take place to prevent PTMs that can inactivate them as seen in the case of unconventional secretion of FGF2 which prevents its *O*-glycosylation that makes it biologically inactive (Wegehingel et al., 2008). Similarly, in another example, it is reported that the conserved LIR motif in connexins mediate the ubiquitin independent binding to the autophagic proteins of LC3 (Catarino et al., 2020). Likewise, the specific phosphorylation of the small heat shock protein, α-Crystallin B at the Ser59 residue enhances autophagy mediated secretion (D’Agostino et al., 2019). The discovery of the diacidic motif, DE as the signal for UCPS (Cruz-Garcia et al., 2017) along with the context dependence of the presence of this motif in proximity with the LIR motif (Padmanabhan et al., 2018) might provide some clues. With the DE as the UCPS export signal, the LIR containing proteins possess specific membrane associated receptors and the cells might use this in combination for the type III secretion. This can be resonated with the hypothesis that the UCPS cargo containing DE binds to specific binding partner (Cruz-Garcia et al., 2018). Probably the presence of LIR motif would recruit the autophagic machinery which would help in the type III mode of autophagy dependent secretion. The role of the protein channel TMED10 in the selective secretion of leaderless cargoes with the help of HSP90s has also helped in understanding the translocation process of UCPS (Liu et al., 2020; Zhang et al., 2020), and the role of the conserved motifs (Motifs 1 and 2) to be the deciding signal in all the UCPS cargoes need to be validated. Thus, the stress adaptive process of autophagy might play a significant role in shifting the existing paradigms and in exploring many autophagy mediated therapeutic interventions (Cotzomi-Ortega et al., 2018; New and Thomas, 2019; Gonzalez et al., 2020).

## Author Contributions

SP: conceptualization, writing – original draft preparation, review, and editing. RM: conceptualization, supervision, writing – review, and editing. Both authors contributed to the article and approved the submitted version.

## Conflict of Interest

The authors declare that the research was conducted in the absence of any commercial or financial relationships that could be construed as a potential conflict of interest.

## References

[B1] An Q. L. Ehlers K. Kogel K. H. van Bel A. J. E. Huckelhoven R. (2006). Multivesicular compartments proliferate in susceptible and resistant MLA12-barley leaves in response to infection by the biotrophic powdery mildew fungus. *New Phytol.* 172 563–576. 10.1111/j.1469-8137.2006.01844.x 17083686

[B2] Anjard C. Loomis W. F. (2005). Peptide signaling during terminal differentiation of *Dictyostelium*. *Proc. Natl. Acad. Sci. U.S.A.* 102 7607–7611. 10.1073/pnas.0501820102 15897458 PMC1140433

[B3] Barlowe C. Orci L. Yeung T. Hosobuchi M. Hamamoto S. Salama N. (1994). Copii – a membrane coat formed by sec proteins that drive vesicle budding from the endoplasmic-reticulum. *Cell* 77 895–907. 10.1016/0092-8674(94)90138-4 8004676

[B4] Berg T. O. Fengsrud M. Stromhaug P. E. Berg T. Seglen P. O. (1998). Isolation and characterization of rat liver amphisomes. Evidence for fusion of autophagosomes with both early and late endosomes. *J. Biol. Chem.* 273 21883–21892. 10.1074/jbc.273.34.21883 9705327

[B5] Bobrie A. Colombo M. Raposo G. Thery C. (2011). Exosome secretion: molecular mechanisms and roles in immune responses. *Traffic* 12 1659–1668. 10.1111/j.1600-0854.2011.01225.x 21645191

[B6] Bonifacino J. S. (2014). Vesicular transport earns a nobel. *Trends Cell Biol.* 24 3–5. 10.1016/j.tcb.2013.11.001 24373306 PMC4788104

[B7] Bruns C. McCaffery J. M. Curwin A. J. Duran J. M. Malhotra V. (2011). Biogenesis of a novel compartment for autophagosome-mediated unconventional protein secretion. *J. Cell Biol.* 195 979–992. 10.1083/jcb.201106098 22144692 PMC3241719

[B8] Burggraaf A. M. Punt P. J. Ram A. F. (2016). The unconventional secretion of PepN is independent of a functional autophagy machinery in the filamentous fungus *Aspergillus niger*. *FEMS Microbiol. Lett.* 363:fnw152. 10.1093/femsle/fnw152 27284019

[B9] Catarino S. Ribeiro-Rodrigues T. M. Sa Ferreira R. Ramalho J. Abert C. Martens S. (2020). A conserved LIR motif in connexins mediates ubiquitin-independent binding to LC3/GABARAP proteins. *Cells* 9:902. 10.3390/cells9040902 32272685 PMC7226732

[B10] Chai X. Dage J. L. Citron M. (2012). Constitutive secretion of tau protein by an unconventional mechanism. *Neurobiol. Dis.* 48 356–366. 10.1016/j.nbd.2012.05.021 22668776

[B11] Chen Y. Toffaletti D. L. Tenor J. L. Litvintseva A. P. Fang C. Mitchell T. G. (2014). The *Cryptococcus neoformans* transcriptome at the site of human meningitis. *Mbio* 5:e01087-13. 10.1128/mBio.01087-13 24496797 PMC3950508

[B12] Cotzomi-Ortega I. Aguilar-Alonso P. Reyes-Leyva J. Maycotte P. (2018). Autophagy and its role in protein secretion: implications for cancer therapy. *Mediators Inflamm.* 2018:4231591. 10.1155/2018/4231591 30622432 PMC6304875

[B13] Cruz-Garcia D. Brouwers N. Duran J. M. Mora G. Curwin A. J. Malhotra V. (2017). A diacidic motif determines unconventional secretion of wild-type and ALS-linked mutant SOD1. *J. Cell Biol.* 216 2691–2700. 10.1083/jcb.201704056 28794127 PMC5584182

[B14] Cruz-Garcia D. Brouwers N. Malhotra V. Curwin A. J. (2020). Reactive oxygen species triggers unconventional secretion of antioxidants and Acb1. *J. Cell Biol.* 219:e201905028. 10.1083/jcb.201905028 32328640 PMC7147093

[B15] Cruz-Garcia D. Malhotra V. Curwin A. J. (2018). Unconventional protein secretion triggered by nutrient starvation. *Semin. Cell Dev. Biol.* 83 22–28. 10.1016/j.semcdb.2018.02.021 29486236

[B16] Curwin A. J. Brouwers N. Adell M. A. Y. Teis D. Turacchio G. Parashuraman S. (2016). ESCRT-III drives the final stages of CUPS maturation for unconventional protein secretion. *Elife* 5:e16299. 10.7554/eLife.16299 27115345 PMC4868542

[B17] D’Agostino M. Scerra G. Serio M. C. Caporaso M. G. Bonatti S. Renna M. (2019). Unconventional secretion of alpha-Crystallin B requires the Autophagic pathway and is controlled by phosphorylation of its serine 59 residue. *Sci. Rep.* 9:16892. 10.1038/s41598-019-53226-x 31729431 PMC6858465

[B18] DeSelm C. J. Miller B. C. Zou W. Beatty W. L. van Meel E. Takahata Y. (2011). Autophagy proteins regulate the secretory component of osteoclastic bone resorption. *Dev. Cell.* 21 966–974. 10.1016/j.devcel.2011.08.016 22055344 PMC3244473

[B19] Dickinson J. D. Alevy Y. Malvin N. P. Patel K. K. Gunsten S. P. Holtzman M. J. (2016). IL13 activates autophagy to regulate secretion in airway epithelial cells. *Autophagy* 12 397–409. 10.1080/15548627.2015.1056967 26062017 PMC4835964

[B20] Dupont N. Jiang S. Y. Pilli M. Ornatowski W. Bhattacharya D. Deretic V. (2011). Autophagy-based unconventional secretory pathway for extracellular delivery of IL-1 beta. *EMBO J.* 30 4701–4711. 10.1038/emboj.2011.398 22068051 PMC3243609

[B21] Duran J. M. Anjard C. Stefan C. Loomis W. F. Malhotra V. (2010). Unconventional secretion of Acb1 is mediated by autophagosomes. *J. Cell Biol.* 188 527–536. 10.1083/jcb.200911154 20156967 PMC2828925

[B22] Ejlerskov P. Rasmussen I. Nielsen T. T. Bergstrom A. L. Tohyama Y. Jensen P. H. (2013). Tubulin polymerization-promoting protein (TPPP/p25alpha) promotes unconventional secretion of alpha-synuclein through exophagy by impairing autophagosome-lysosome fusion. *J. Biol. Chem.* 288 17313–17335. 10.1074/jbc.M112.401174 23629650 PMC3682534

[B23] Fader C. M. Colombo M. I. (2009). Autophagy and multivesicular bodies: two closely related partners. *Cell Death Differ.* 16 70–78. 10.1038/cdd.2008.168 19008921

[B24] Filimonenko M. Stuffers S. Raiborg C. Yamamoto A. Malerod L. Fisher E. M. (2007). Functional multivesicular bodies are required for autophagic clearance of protein aggregates associated with neurodegenerative disease. *J. Cell Biol.* 179 485–500. 10.1083/jcb.200702115 17984323 PMC2064794

[B25] Fujii K. Munshi U. M. Ablan S. D. Demirov D. G. Soheilian F. Nagashima K. (2009). Functional role of Alix in HIV-1 replication. *Virology* 391 284–292. 10.1016/j.virol.2009.06.016 19596386 PMC2744943

[B26] Gee H. Y. Noh S. H. Tang B. L. Kim K. H. Lee M. G. (2011). Rescue of DeltaF508-CFTR trafficking via a GRASP-dependent unconventional secretion pathway. *Cell* 146 746–760. 10.1016/j.cell.2011.07.021 21884936

[B27] Godinho R. M. D. Crestani J. Kmetzsch L. Araujo G. D. Frases S. Staats C. C. (2014). The vacuolar-sorting protein Snf7 is required for export of virulence determinants in members of the *Cryptococcus neoformans* complex. *Sci. Rep.* 4:6198 10.1038/sre06198 PMC4151102 25178636

[B28] Gonzalez C. D. Resnik R. Vaccaro M. (2020). Secretory autophagy and its relevance in metabolic and degenerative disease. *Front. Endocrinol.* 11:266. 10.3389/fendo.2020.00266 32477265 PMC7232537

[B29] Hata Y. Slaughter C. A. Sudhof T. C. (1993). Synaptic vesicle fusion complex contains unc-18 homologue bound to syntaxin. *Nature* 366 347–351. 10.1038/366347a0 8247129

[B30] Hessvik N. P. Overbye A. Brech A. Torgersen M. L. Jakobsen I. S. Sandvig K. (2016). PIKfyve inhibition increases exosome release and induces secretory autophagy. *Cell. Mol. Life Sci.* 73 4717–4737. 10.1007/s00018-016-2309-8 27438886 PMC11108566

[B31] Hirsch J. G. Fedorko M. E. Cohn Z. A. (1968). Vesicle fusion and formation at the surface of pinocytic vacuoles in macrophages. *J. Cell Biol.* 38 629–632. 5664229 10.1083/jcb.38.3.629 PMC2108378

[B32] Ishibashi K. Uemura T. Waguri S. Fukuda M. (2012). Atg16L1, an essential factor for canonical autophagy, participates in hormone secretion from PC12 cells independently of autophagic activity. *Mol. Biol. Cell* 23 3193–3202. 10.1091/mbc.E12-01-0010 22740627 PMC3418313

[B33] Jiang S. Dupont N. Castillo E. F. Deretic V. (2013). Secretory versus degradative autophagy: unconventional secretion of inflammatory mediators. *J. Innate Immun.* 5 471–479. 10.1159/000346707 23445716 PMC3723810

[B34] Josephrajan A. Hertzel A. V. Bohm E. K. McBurney M. W. Imai S. I. Mashek D. G. (2019). Unconventional secretion of adipocyte fatty acid binding protein 4 is mediated by autophagic proteins in a sirtuin-1-dependent manner. *Diabetes* 68 1767–1777. 10.2337/db18-1367 31171562 PMC6702637

[B35] Julenius K. Pedersen A. G. (2006). Protein evolution is faster outside the cell. *Mol. Biol. Evol.* 23 2039–2048. 10.1093/molbev/msl081 16891379

[B36] Katsinelos T. Zeitler M. Dimou E. Karakatsani A. Muller H. M. Nachman E. (2018). Unconventional secretion mediates the trans-cellular spreading of Tau. *Cell Rep.* 23 2039–2055. 10.1016/j.celrep.2018.04.056 29768203

[B37] Kim J. Noh S. H. Piao H. Kim D. H. Kim K. Cha J. S. (2016). Monomerization and ER relocalization of GRASP is a requisite for unconventional secretion of CFTR. *Traffic* 17 733–753. 10.1111/tra.12403 27062250

[B38] Kimura T. Jia J. Kumar S. Choi S. W. Gu Y. Mudd M. (2017). Dedicated SNAREs and specialized TRIM cargo receptors mediate secretory autophagy. *EMBO J.* 36 42–60. 10.15252/embj.201695081 27932448 PMC5210154

[B39] Kinseth M. A. Anjard C. Fuller D. Guizzunti G. Loomis W. F. Malhotra V. (2007). The golgi-associated protein GRASP is required for unconventional protein secretion during development. *Cell* 130 524–534. 10.1016/j.cell.2007.06.029 17655921

[B40] Kortvely E. Hauck S. M. Behler J. Ho N. Ueffing M. (2016). The unconventional secretion of ARMS2. *Hum. Mol. Genet.* 25 3143–3151. 10.1093/hmg/ddw162 27270414

[B41] Leidal A. M. Debnath J. (2020). LC3-dependent extracellular vesicle loading and secretion (LDELS). *Autophagy* 16 1162–1163. 10.1080/15548627.2020.1756557 32330402 PMC7469500

[B42] Leidal A. M. Huang H. H. Marsh T. Solvik T. Zhang D. Ye J. (2020). The LC3-conjugation machinery specifies the loading of RNA-binding proteins into extracellular vesicles. *Nat. Cell Biol.* 22 187–199. 10.1038/s41556-019-0450-y 31932738 PMC7007875

[B43] Li Y. D. Xie Z. Y. Du Y. L. Zhou Z. Mao X. M. Lv L. X. (2009). The rapid evolution of signal peptides is mainly caused by relaxed selection on non-synonymous and synonymous sites. *Gene* 436 8–11. 10.1016/j.gene.2009.01.015 19393172

[B44] Liu L. Zhang M. Ge L. (2020). Protein translocation into the ERGIC: an upstream event of secretory autophagy. *Autophagy* 16 1358–1360. 10.1080/15548627.2020.1768668 32521187 PMC7469677

[B45] Loomis W. F. Behrens M. M. Williams M. E. Anjard C. (2010). Pregnenolone sulfate and cortisol induce secretion of acyl-CoA-binding protein and its conversion into endozepines from astrocytes. *J. Biol. Chem.* 285 21359–21365. 10.1074/jbc.M110.105858 20452969 PMC2898429

[B46] Maiuri L. Kroemer G. (2018). Autophagy delays progression of the two most frequent human monogenetic lethal diseases: cystic fibrosis and Wilson disease. *Aging* 10 3657–3661. 30568028 10.18632/aging.101736 PMC6326686

[B47] Maiuri L. Raia V. Piacentini M. Tosco A. Villella V. R. Kroemer G. (2019). Cystic fibrosis transmembrane conductance regulator (CFTR) and autophagy: hereditary defects in cystic fibrosis versus gluten-mediated inhibition in celiac disease. *Oncotarget* 10 4492–4500. 10.18632/oncotarget.27037 31321000 PMC6633896

[B48] Malhotra V. (2013). Unconventional protein secretion: an evolving mechanism. *EMBO J.* 32 1660–1664. 10.1038/emboj.2013.104 23665917 PMC3680731

[B49] Manjithaya R. Anjard C. Loomis W. F. Subramani S. (2010). Unconventional secretion of Pichia pastoris Acb1 is dependent on GRASP protein, peroxisomal functions, and autophagosome formation. *J. Cell Biol.* 188 537–546. 10.1083/jcb.200911149 20156962 PMC2828923

[B50] Manjithaya R. Subramani S. (2010). Role of autophagy in unconventional protein secretion. *Autophagy* 6 650–651. 10.4161/auto.6.5.12066 20473033 PMC3677939

[B51] Manjithaya R. Subramani S. (2011). Autophagy: a broad role in unconventional protein secretion? *Trends Cell Biol.* 21 67–73. 10.1016/j.tcb.2010.09.009 20961762 PMC3025270

[B52] McCotter S. W. Horianopoulos L. C. Kronstad J. W. (2016). Regulation of the fungal secretome. *Curr. Genet.* 62 533–545. 10.1007/s00294-016-0578-2 26879194

[B53] Mobius W. Ohno-Iwashita Y. van Donselaar E. G. Oorschot V. M. Shimada Y. Fujimoto T. (2002). Immunoelectron microscopic localization of cholesterol using biotinylated and non-cytolytic perfringolysin O. *J. Histochem. Cytochem.* 50 43–55. 11748293 10.1177/002215540205000105

[B54] Mohamed N. V. Plouffe V. Remillard-Labrosse G. Planel E. Leclerc N. (2014). Starvation and inhibition of lysosomal function increased tau secretion by primary cortical neurons. *Sci. Rep.* 4 5715. 10.1038/srep05715 25030297 PMC4101526

[B55] Murrow L. Malhotra R. Debnath J. (2015). ATG12-ATG3 interacts with Alix to promote basal autophagic flux and late endosome function. *Nat. Cell Biol.* 17 300–310. 10.1038/ncb3112 25686249 PMC4344874

[B56] Narita M. Young A. R. Arakawa S. Samarajiwa S. A. Nakashima T. Yoshida S. (2011). Spatial coupling of mTOR and autophagy augments secretory phenotypes. *Science* 332 966–970. 10.1126/science.1205407 21512002 PMC3426290

[B57] New J. Thomas S. M. (2019). Autophagy-dependent secretion: mechanism, factors secreted, and disease implications. *Autophagy* 15 1682–1693. 10.1080/15548627.2019.1596479 30894055 PMC6735501

[B58] Nilsson P. Loganathan K. Sekiguchi M. Matsuba Y. Hui K. Tsubuki S. (2013). Abeta secretion and plaque formation depend on autophagy. *Cell Rep.* 5 61–69. 10.1016/j.celrep.2013.08.042 24095740

[B59] Noh S. H. Gee H. Y. Kim Y. Piao H. Kim J. Kang C. M. (2018). Specific autophagy and ESCRT components participate in the unconventional secretion of CFTR. *Specific Autophagy* 14 1761–1778. 10.1080/15548627.2018.1489479 29969945 PMC6135621

[B60] Nuchel J. Ghatak S. Zuk A. V. Illerhaus A. Morgelin M. Schonborn K. (2018). TGFB1 is secreted through an unconventional pathway dependent on the autophagic machinery and cytoskeletal regulators. *Autophagy* 14 465–486. 10.1080/15548627.2017.1422850 29297744 PMC5915026

[B61] Ohman T. Teirila L. Lahesmaa-Korpinen A. M. Cypryk W. Veckman V. Saijo S. (2014). Dectin-1 pathway activates robust autophagy-dependent unconventional protein secretion in human macrophages. *J. Immunol.* 192 5952–5962. 10.4049/jimmunol.1303213 24808366

[B62] Padmanabhan S. Biswal M. R. Manjithaya R. Prakash M. K. (2018). Exploring the context of diacidic motif DE as a signal for unconventional protein secretion in eukaryotic proteins. *Wellcome Open Res.* 3:148. 10.12688/wellcomeopenres.14914.1 30607372 PMC6305234

[B63] Pilli M. Arko-Mensah J. Ponpuak M. Roberts E. Master S. Mandell M. A. (2012). TBK-1 promotes autophagy-mediated antimicrobial defense by controlling autophagosome maturation. *Immunity* 37 223–234. 10.1016/j.immuni.2012.04.015 22921120 PMC3428731

[B64] Rabouille C. (2016). Pathways of unconventional protein secretion. *Trends Cell Biol.* 27 230–240. 10.1016/j.tcb.2016.11.007 27989656

[B65] Rabouille C. Malhotra V. Nickel W. (2012). Diversity in unconventional protein secretion. *J. Cell Sci.* 125(Pt 22) 5251–5255. 10.1242/jcs.103630 23377655

[B66] Rubinsztein D. C. Codogno P. Levine B. (2012). Autophagy modulation as a potential therapeutic target for diverse diseases. *Nat. Rev. Drug Discov.* 11 709–U784. 10.1038/nrd3802 22935804 PMC3518431

[B67] Schotman H. Karhinen L. Rabouille C. (2008). dGRASP-mediated noncanonical integrin secretion is required for *Drosophila* epithelial remodeling. *Dev. Cell* 14 171–182. 10.1016/j.devcel.2007.12.006 18267086

[B68] Simon D. Garcia-Garcia E. Gomez-Ramos A. Falcon-Perez J. M. Diaz-Hernandez M. Hernandez F. (2012). Tau overexpression results in its secretion via membrane vesicles. *Neurodegener. Dis.* 10 73–75. 10.1159/000334915 22269430

[B69] Sollner T. Whiteheart S. W. Brunner M. Erdjument-Bromage H. Geromanos S. Tempst P. (1993). SNAP receptors implicated in vesicle targeting and fusion. *Nature* 362 318–324. 10.1038/362318a0 8455717

[B70] Son S. M. Cha M. Y. Choi H. Kang S. Choi H. Lee M. S. (2016). Insulin-degrading enzyme secretion from astrocytes is mediated by an autophagy-based unconventional secretory pathway in Alzheimer disease. *Autophagy* 12 784–800. 10.1080/15548627.2016.1159375 26963025 PMC4854558

[B71] Son S. M. Kang S. Choi H. Mook-Jung I. (2015). Statins induce insulin-degrading enzyme secretion from astrocytes via an autophagy-based unconventional secretory pathway. *Mol. Neurodegener.* 10:56. 10.1186/s13024-015-0054-3 26520569 PMC4628355

[B72] Suh J. Hutter H. (2012). A survey of putative secreted and transmembrane proteins encoded in the C. elegans genome. *BMC Genomics* 13:333. 10.1186/1471-2164-13-333 22823938 PMC3534327

[B73] Thakur S. Normand P. Daubin V. Tisa L. S. Sen A. (2013). Contrasted evolutionary constraints on secreted and non-secreted proteomes of selected Actinobacteria. *BMC Genomics* 14:474. 10.1186/1471-2164-14-474 23848577 PMC3729583

[B74] Torisu T. Torisu K. Lee I. H. Liu J. Malide D. Combs C. A. (2013). Autophagy regulates endothelial cell processing, maturation and secretion of von Willebrand factor. *Nat. Med.* 19 1281–1287. 10.1038/nm.3288 24056772 PMC3795899

[B75] Tsuboi K. Nishitani M. Takakura A. Imai Y. Komatsu M. Kawashima H. (2015). Autophagy protects against colitis by the maintenance of normal gut microflora and secretion of mucus. *J. Biol. Chem.* 290 20511–20526. 10.1074/jbc.M114.632257 26149685 PMC4536456

[B76] Villeneuve J. Bassaganyas L. Lepreux S. Chiritoiu M. Costet P. Ripoche J. (2018). Unconventional secretion of FABP4 by endosomes and secretory lysosomes. *J. Cell Biol.* 217 649–665. 10.1083/jcb.201705047 29212659 PMC5800802

[B77] Viotti C. (2016). ER to golgi-dependent protein secretion: the conventional pathway. *Methods Mol. Biol.* 1459 3–29. 10.1007/978-1-4939-3804-9_1 27665548

[B78] Wegehingel S. Zehe C. Nickel W. (2008). Rerouting of fibroblast growth factor 2 to the classical secretory pathway results in post-translational modifications that block binding to heparan sulfate proteoglycans. *FEBS Lett.* 582 2387–2392. 10.1016/j.febslet.2008.05.042 18538671

[B79] Williams E. J. Pal C. Hurst L. D. (2000). The molecular evolution of signal peptides. *Gene* 253 313–322. 10940569 10.1016/s0378-1119(00)00233-x

[B80] Winter E. E. Goodstadt L. Ponting C. P. (2004). Elevated rates of protein secretion, evolution, and disease among tissue-specific genes. *Genome Res.* 14 54–61. 10.1101/gr.1924004 14707169 PMC314278

[B81] Xu X. Zhao Y. Kirkman E. Lin X. (2015). Secreted Acb1 contributes to the yeast-to-hypha transition in *Cryptococcus neoformans*. *Appl. Environ. Microbiol.* 82 1069–1079. 10.1128/AEM.03691-15 26637591 PMC4751841

[B82] Yamamoto A. Cremona M. L. Rothman J. E. (2006). Autophagy-mediated clearance of huntingtin aggregates triggered by the insulin-signaling pathway. *J. Cell Biol.* 172 719–731. 10.1083/jcb.200510065 16505167 PMC2063704

[B83] Yin X. Zhou C. Li J. Liu R. Shi B. Yuan Q. (2019). Autophagy in bone homeostasis and the onset of osteoporosis. *Bone Res.* 7:28. 10.1038/s41413-019-0058-7 31666998 PMC6804951

[B84] Zhang M. Kenny S. J. Ge L. Xu K. Schekman R. (2015). Translocation of interleukin-1β into a vesicle intermediate in autophagy-mediated secretion. *eLife* 4:e11205. 10.7554/eLife.11205 26523392 PMC4728131

[B85] Zhang M. Liu L. Lin X. Wang Y. Li Y. Guo Q. (2020). A translocation pathway for vesicle-mediated unconventional protein secretion. *Cell* 181 637–652.e615. 10.1016/j.cell.2020.03.031 32272059

